# Synergistic combination therapy using cowpea mosaic virus intratumoral immunotherapy and Lag-3 checkpoint blockade

**DOI:** 10.1007/s00262-024-03636-2

**Published:** 2024-02-13

**Authors:** Sweta Karan, Eunkyeong Jung, Christine Boone, Nicole F. Steinmetz

**Affiliations:** 1 Department of Nanoengineering, University of California, San Diego, La Jolla, CA USA; 2 Department of Radiology, University of California, San Diego, La Jolla, CA USA; 3 Shu and K.C. Chien and Peter Farrell Collaboratory, University of California, San Diego, La Jolla, CA USA; 4 Center for Nano-ImmunoEngineering, University of California, San Diego, La Jolla, CA USA; 5 Moores Cancer Center, University of California, San Diego, La Jolla, CA USA; 6 Department of Bioengineering, University of California, San Diego, La Jolla, CA USA; 7 Institute for Materials Discovery and Design, University of California, San Diego, La Jolla, CA USA; 8 Center for Engineering in Cancer, Institute of Engineering Medicine, University of California, San Diego, La Jolla, CA USA

**Keywords:** Intratumoral immunotherapy, Cowpea mosaic virus, Checkpoint therapy, Lag-3

## Abstract

Immune checkpoint therapy (ICT) for cancer can yield dramatic clinical responses; however, these may only be observed in a minority of patients. These responses can be further limited by subsequent disease recurrence and resistance. Combination immunotherapy strategies are being developed to overcome these limitations. We have previously reported enhanced efficacy of combined intratumoral cowpea mosaic virus immunotherapy (CPMV IIT) and ICT approaches. Lymphocyte-activation gene-3 (LAG-3) is a next-generation inhibitory immune checkpoint with broad expression across multiple immune cell subsets. Its expression increases on activated T cells and contributes to T cell exhaustion. We observed heightened efficacy of a combined CPMV IIT and anti-LAG-3 treatment in a mouse model of melanoma. Further, LAG-3 expression was found to be increased within the TME following intratumoral CPMV administration. The integration of CPMV IIT with LAG-3 inhibition holds significant potential to improve treatment outcomes by concurrently inducing a comprehensive anti-tumor immune response, enhancing local immune activation, and mitigating T cell exhaustion.

## Introduction

Immunotherapy has rapidly been integrated into first- and second-line treatment for numerous cancers, including melanoma, lung cancer, and gastrointestinal malignancies. The remarkable efficacy of first-generation immune checkpoint therapy (ICT), which antagonize inhibitory receptors, cytotoxic T-lymphocyte-associated antigen-4 (CTLA-4) or programmed cell death-(ligand)1 (PD-(L)1), became the driving force behind this paradigm shift. As ICT applications expanded and longer-term outcomes were evaluated, it became clear that the first-generation ICT only demonstrated efficacy in a minority of patients [1–5]. Over time, patients with initial responses developed recurrent and resistant disease, particularly those with solid tumors [6]. To overcome these limitations, preclinical and clinical development of immunotherapeutic strategies has emphasized combination approaches. These include multiple ICTs and multiple modalities, such as radiation and ICT [7] or chemotherapy and ICT [8, 9]. These combination approaches have yielded improved clinical responses over monotherapy alone. On-going discovery and development of other immunotherapeutic strategies, as well as the next generation of ICTs further fuel opportunity for novel combination approaches in immunotherapy [10, 11]. With the expansive and continually expanding range of therapeutic approaches, there is a need for preclinical data to help inform combinations put to clinical trial. In this work, we aimed to determine the efficacy of a novel immunotherapeutic approach in combination with a second-generation ICT.

Intratumoral immunotherapy (IIT) is a distinctive strategy, entailing the direct introduction of an adjuvant or immunostimulatory agent into a tumor. IIT can induce an adaptive immune response, which not only generates a systemic, targeted response to the tumor, but can also lead to immunological memory against the tumor. This response is mediated by polyclonal effector T cells. IIT can also alter the tumor microenvironment (TME) to facilitate the anti-tumor immune response. The TME is often immunosuppressive or “cold”, comprising of immune checkpoints, pro-tumor cytokines, and recruitment of tumor-associated macrophages (TAMs), T cells (Tregs) and myeloid-derived suppressor cells (MDSCs) [12, 13]. It is a major physical and biological obstacle to anti-tumor immune responses in solid tumors, even when there is evidence of a systemic adaptive response against the tumor [14–16]. Furthermore, IIT does not require exogenous targeting or modification of the therapy for each patient. Multiple strategies, including injection of monoclonal antibody (mAb), CAR-T cells, bacterial vectors, and viral vectors, have been employed in preclinical and clinical studies [17–21]. Clinical trials have revealed survival benefit in treatment of advanced stage and metastatic melanoma [22, 23] and hepatocellular carcinoma [24] with genetically modified oncolytic viruses administered intratumorally.

IIT with cowpea mosaic virus (CPMV), a plant virus, as the adjuvant has demonstrated distinctive efficacy across multiple murine model tumor types, including melanoma [25–27], ovarian [28, 29], colorectal [25], glioma [30], and breast [25] cancers, as well as in canine cancer patients with sporadic tumors [31, 32]. Intratumorally administered CPMV induces a systemic and durable immunological anti-tumor response with immunological memory to prevent recurrence. Further, while CPMV is efficacious as a monotherapy, its efficacy is augmented in combination with radiation [33], chemotherapy [34], and checkpoint inhibitors [35].

In this and our previous work, a “live” CPMV particle containing its bipartite RNA genome is used. CPMV is a non-enveloped positive sense RNA plant virus. “Live” CPMV is infectious toward plants, including black-eyed peas and other legumes, there are, however, no reports indicating CPMV to infect or replicate in mammals. Therefore, this biologic drug candidate is distinct from oncolytic viruses that infect, replicate, and express proteins in target tumor cells [36]. CPMV IIT can relieve immunosuppression in the TME and prime systemic anti-tumor immunity [37, 38]. CPMV IIT interacts with the immune system in a multivalent manner, resulting in a cascade of events boosted by avidity to achieve unprecedented potency. CPMV binds activates multiple toll-like receptors (TLRs), TLR2, TLR4, and TLR7 [39–41]. Intratumoral CPMV primes the TME to become immunostimulatory by regulating the phenotypes of tumor-resident and infiltrated macrophages, promoting conversion from M2 to M1 types, expansion of plasmacytoid dendritic cells (pDCs), and the infiltration and activation of N1-type neutrophils and natural killer (NK) cells. These activated and mature innate immune cells process tumor-associated antigens and neoantigens in the tumor to generate tumor-specific CD4^+^ and CD8^+^ effector and memory T cells [26, 28, 42, 43]. In previous work, we found that combined CPMV IIT with checkpoint therapy targeting PD1, OX40, or 4-1BB augmented potency beyond each as a single-agent treatment. [35, 44]

In this work, we focused on another combination approach utilizing CPMV IIT with a next-generation ICT, lymphocyte activation gene-3 (LAG-3) inhibition. Structurally, LAG-3 possesses unique intracellular structural motifs suggesting a distinctive mechanism of action compared to other immune checkpoints. While its signaling mechanisms and the extent of its expression patterns remain an active area of investigation, LAG-3 is often implicated in tumor-mediated immunosuppression and immune cell homeostasis [45–47]. LAG-3 acts as a co-inhibitory receptor to major histocompatibility complex class II (MHCII) on the surface of T cells. LAG-3 binds to MHCII molecules with higher affinity than CD4^+^. When engaged, it inhibits T cell activation, proliferation, and cytokine production. LAG-3 expression increases on activated T cells and contributes to T cell exhaustion. Proliferation of activated CD4^+^ T cells and their production of IL2, IL-4, IFN-γ, and TNFα were enhanced with LAG-3 blockade [48]. Therefore, the LAG-3 MHCII binding can inhibit the activation of CD4^+^ T cells and negatively regulate their function [46–50]. LAG-3 is expressed on Tregs and can enhance their suppressive function, leading to a more immunosuppressive TME [51]. LAG-3 is also expressed on NK cells and contributes to their regulation of their function. Blocking LAG-3 can restore NK cell-mediated cytotoxicity against tumor cells as well as the function of exhausted T cells and enhance anti-tumor immunity [45–47]. LAG-3 is also constitutively and most highly expressed on plasmacytoid dendritic cells (pDCs). pDCs are key in initiation of immune responses, but in the context of cancer they are also tolerogenic. [52]

LAG-3 inhibition has shown potential as a cancer ICT in both clinical and preclinical studies. Early clinical trials have shown safety and tolerability of LAG-3 inhibition in combination with chemotherapy [53–56]. Durable clinical responses, increased progression free-survival was also seen in combinations of anti-PD1 and Lag-3 inhibition [57, 58]. Both intratumoral CPMV therapy and LAG-3 inhibition act on a multiple immune cell types and pathways to promote an anti-tumor immune response. We therefore, investigated the combination of CPMV IIT and LAG-3 blockade to treat an aggressive and immunosuppressive mouse model of melanoma, B16F10 [59]. We also examined how CPMV IIT combination treatment altered the tumor microenvironment with respect to LAG-3 expression.

## Methods

### CPMV purification and characterization

CPMV was propagated in black-eyed pea no. 5 plants and purified as previously reported [60]. The concentration of the purified CPMV fraction was determined by UV–Vis spectroscopy ((ε of CPMV at 260 nm = 8.1 mL / (mg x cm)), and particle integrity was confirmed by NuPAGE, transmission electron microscopy (TEM), dynamic light scattering (DLS), and size exclusion chromatography (SEC). For NuPAGE analysis, 10 µg heat-denatured samples (mixed with 4 × lithium dodecylsulfate buffer, Thermo Fisher Scientific) were analyzed using a 4–12% NuPAGE gel in 1 × MOPS buffer (Thermo Fisher Scientific). Gels were stained with Coomassie brilliant blue and imaged using a ProteinSimple FluorChem R imager. For TEM, CPMV (2 µg) was applied to a glow-discharged carbon-coated 300-mesh Cu grid and stained with 4 µL 1% (w/v) uranyl acetate (Electron Microscopy Sciences). The samples were imaged using a Talos TEM (Thermo Fisher Scientific) at a nominal magnification of 120,000 ×  DLS measurements were recorded using a Zetasizer Nano ZSP/Zen5600 instrument (Malvern Panalytical) and 100 µg CPMV (1 mg/mL). For SEC, we used a Superose 6 increase 10/300 GL column mounted on an ÄKTA purifier system (GE Healthcare). 100 µg CPMV (1 mg/mL) was analyzed at a flow rate of 0.5 mL/min, and the absorbance was monitored at 260 nm (RNA) and 280 nm (protein).

### B16F10 melanoma tumor model and efficacy study

B16F10 tumor cells were cultured in Dulbecco's Modified Eagle's Medium containing fetal bovine serum at a final concentration of 10% by volume and supplemented with 1% (v/v) penicillin streptomycin. All mouse studies were carried out in accordance with the guidelines of the Institutional Animal Care and Use Committee (IACUC) of the University of California, San Diego (UCSD), and were approved by the Animal Ethics Committee of UCSD. We obtained 7-week-old female C57BL/6 J mice from Jackson Laboratories. C57BL/6 mice (*n* = 7–9) were intradermally implanted with B16F10 tumor cells (2 × 10^5^ cells). Tumor volume was measured using digital calipers and reported as [length x (short width)2]/2. Treatment began when tumor volume was reached between 40 and 60 mm^3^. Animals were randomly assigned the following groups: CPMV (100 µg), anti-LAG-3 monoclonal antibody (anti-LAG-3, C9B7W, IgG1, BioXcell; 100 µg), CPMV (100 µg) + anti-LAG-3 (100 µg), Control mice were intratumoral injected with PBS. CPMV was administered intratumorally and anti-LAG-3 was injected intraperitoneally (i.p.) on day 7, 15, and 21. Treatment efficacy is measured in terms of delayed tumor growth and overall survival. Treatment studies were repeated with reproducible results.

### Expression profile of LAG-3 in tumors upon CPMV treatment

To examine the expression profile of LAG-3, B16F10 tumor-bearing C57BL/6 mice were intradermally treated with CPMV or PBS when tumors were palpable (40–60 mm^3^). Tumors were collected on day 1, 5, and 10 post-CPMV treatment. Tumor dissociation and single-cell suspensions were obtained using a tumor dissociation kit (Miltenyi Biotec). LAG-3 expression was measured by flow cytometry or confocal imaging. Cells were washed in cold PBS containing 2% (v/v) FBS. Fc receptors were blocked using anti-mouse CD16/CD32 (Biolegend) for 20 min at 4 °C (1:1000). Then staining was performed using anti-LAG-3 monoclonal antibody (CD223 (LAG-3), eBioC9B7W, 0.5 µg) for 2 h at RT. After washing with PBS for three times (in 10 min intervals), cells were stained with secondary goat anti-rat IgG (H + L) antibody conjugated with Alexa Fluor™ Plus 488 (1:1000, 45 min, RT). The stained cells were fixed in biological fixative solution (2% (v/v) formaldehyde in PBS) for 10 min at RT. Flow cytometry was carried out using a using a BD Accuri C6 Plus flow cytometer (BD Biosciences), and the data were analyzed using FlowJo software. For confocal microscopy, B16F10 tumors were snap frozen in liquid nitrogen and stored at –80 °C. The tumors were then submerged in Tissue-Tek™ O.C.T. Compound (Sakura) and cryosectioned using the Leica CM1860 cryostat. Ten µm sections were collected on Superfrost™ Plus Microscope Slides (Fisherbrand) and placed at –80 °C. Staining of tissue section was carried out using primary rat anti-mouse CD45 (cell signaling clone 30-F11; 1:800), anti-LAG-3 (eBioC9B7W, 0.5 µg), and custom-made rabbit anti-CPMV antibodies (1:1000) for 2 h at RT followed by labeling with secondary antibodies for 45 min using goat anti-rat IgG (H&L) conjugated with Alexa Fluor™ 555 (1:1000), anti-rat IgG (H&L) conjugated with Alexa Fluor™ 488 (1:1000), and goat anti-rabbit IgG (H&L) conjugated with Alexa Fluor™ 647 (1:1000), respectively. All slides were then stained and mounted using Fluoroshield with DAPI (Millipore Sigma). Fluorescence images were obtained using a Nikon A1R confocal microscope with an Apo TIRF 100 × /1.49 oil objective (Nikon). Collected images were analyzed using NIS-Elements AR Analysis v5.30 (Nikon).

## Results

**Fig. 1:**
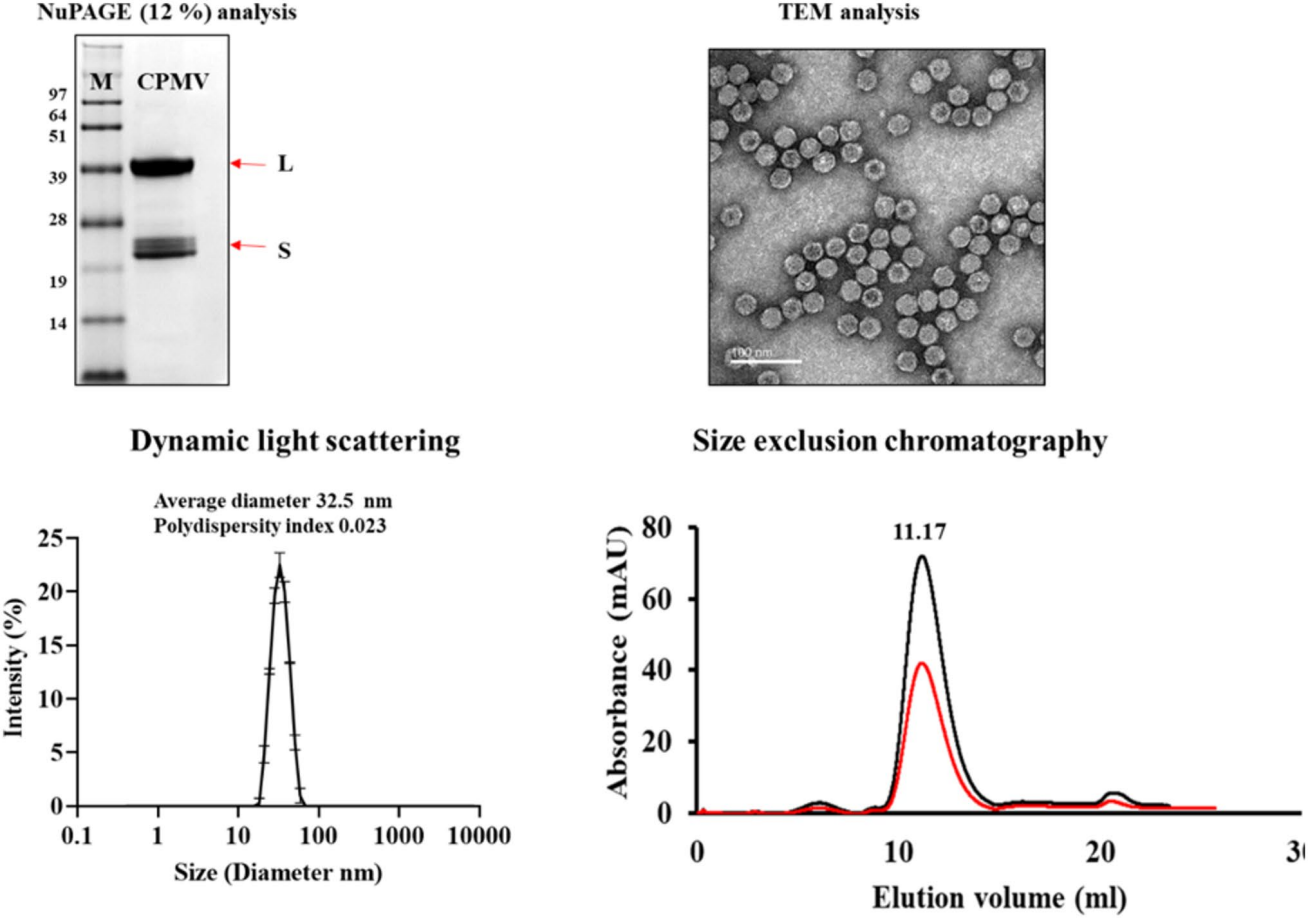
Purification and characterization of cowpea mosaic virus (CPMV) by NuPAGE denaturing SDS-PAGE (4–12%) of the coat proteins stained with Coomassie blue (top left), TEM of purified CPMV (negatively stained with 1% (w/v) uranyl acetate, top right), DLS (bottom left), and SEC (bottom right)

CPMV was purified from black-eyed pea plants at yields of ~ 35 mg per 100 mg of infected leaf tissue. Purity and structural integrity of purified CPMV was validated as follows (Fig. 1): UV–visible spectroscopy was used to determine the concentration and the A260/280 nm ratio of ~ 1.75 indicated that pure CPMV preparations were obtained. NuPAGE analysis showed the small (S) and large (L) coat proteins at ~ 24 kDa and ~ 42 kDa, respectively; protein contaminants were not apparent. Structural integrity was further confirmed by TEM, which demonstrated monodispersed CPMV particles. DLS demonstrated a sharp size distribution of purified CPMV with an average particle size of 32.5 nm (PDI 0.023), consistent with a uniform population of particles. SEC showed the typical elution profile of CPMV at 11.17 mL from the Super 6 increase column; the overlapping peak of CPMV genomic RNA (260 nm) and capsid protein (280 nm) was also consistent with intact CPMV. SEC did not indicate the presence of aggregated or broken particles.

Efficacy of the CPMV IIT + anti-LAG-3 ICT combination was investigated against a dermal B16F10 melanoma mouse model (C67BL/6 J) mice. Female mice were inoculated i.d. with 2 × 10^5^ B16F10 cells on the right flank and then randomized to one of four treatment groups (*n* = 7–9 per group): 100 µg intratumoral (i.t.) CPMV, 100 µg i.p. anti-LAG-3, i.t. CPMV + i.p. anti-LAG-3, or PBS (control). Treatment began when tumors reached 40–60 mm^3^. Mice received doses on post-inoculation days 6, 11, and 16 (it is noted that animals in the PBS and anti-LAG-3 groups were already removed at day 16 based on humane endpoint defined at tumors reaching 1000 mm^3^).

**Fig. 2:**
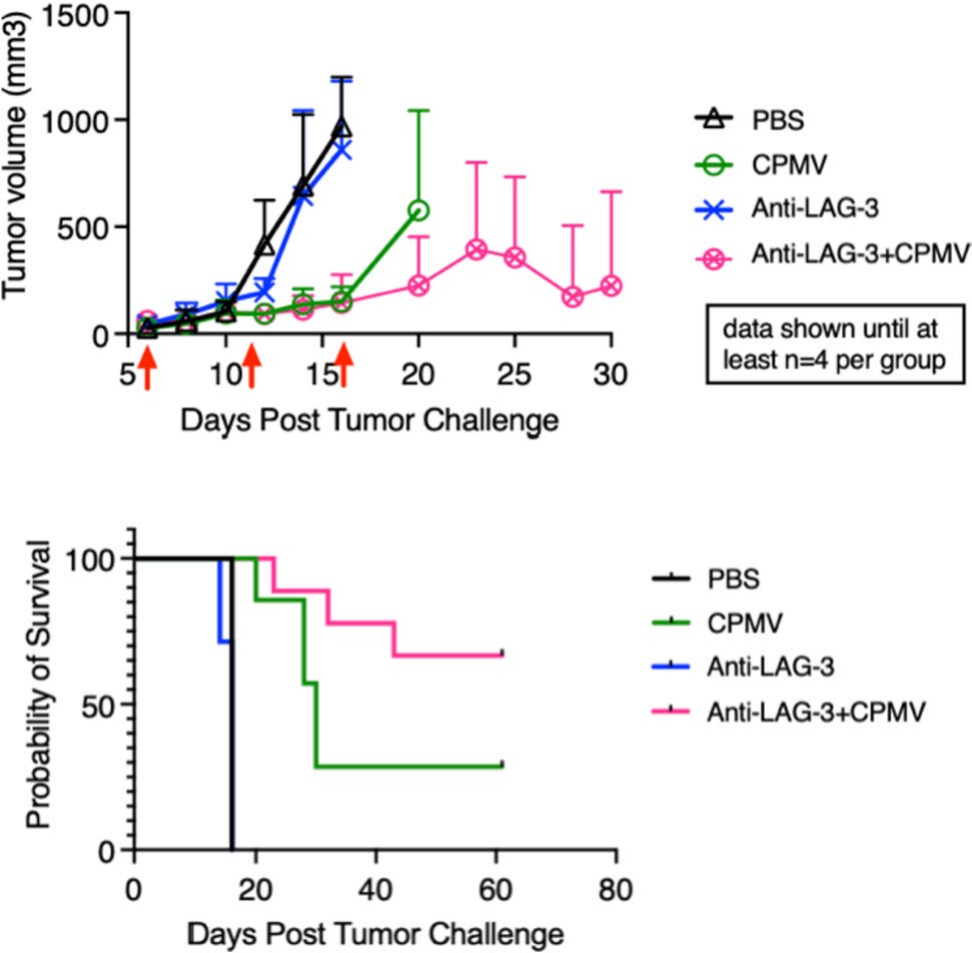
Treatment of B16F10 dermal melanoma using CPMV + anti-LAG-3 antibody therapy. C67BL/6 mice bearing dermal B16F10 melanoma (*n* = 7–9) received 100 µg intratumoral CPMV (green), 100 µg intraperitoneal anti-LAG-3 monoclonal antibody (mAb) (blue), CPMV + anti-LAG-3 mAb (pink), or PBS (control) (black). Treatment began when tumors reached 40–60 mm^3^ and mice received three doses on day 6, 11, and 16 (as indicated by red arrows). *Top panel,* Estimated tumor volume as calculated by volume = [(short length)2 × (long length)]/2. *Bottom panel*, Survival of treatment groups was plotted and statistical analysis was performed using a log-rank (Mantel–Cox) test (anti-Lag-3 + CPMV vs. PBS ****p < 0.0001 and CPMV vs. PBS ***p = *0.0003*

Outcomes of the efficacy study were demonstrated in tumor growth and survival curves (Fig. 2). Combined i.t. CPMV + i.p. anti-LAG-3 ICT was the most potent approach, with the most effective control of tumor growth and 66% survival rate at 60 days post-inoculation (study endpoint). Intratumoral CPMV alone was also efficacious and controlled tumor growth, although to a lesser extent than the combined approach, with 29% of mice surviving at 60 days. This is consistent with our previous observation [25]. Finally, anti-LAG-3 ICT alone conferred no efficacy against B16F10 melanoma. Both PBS and anti-LAG-3 mAb treatment resulted in 100% mortality by day 16 in both groups.

**Fig. 3:**
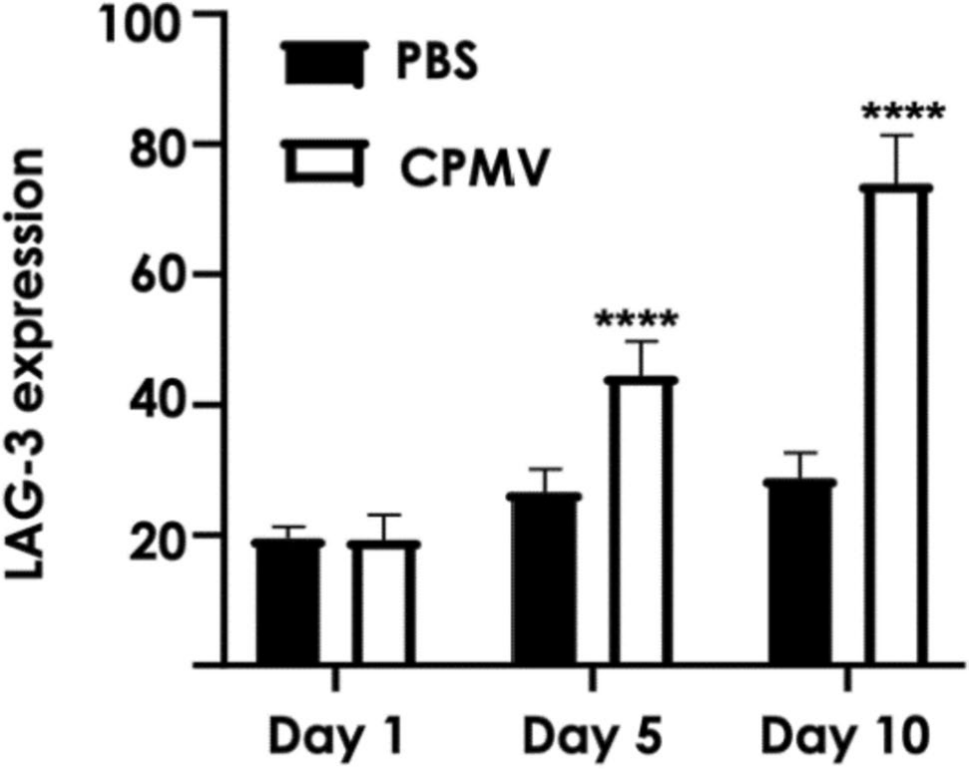
LAG-3 expression on cell suspensions from B16F10 dermal melanomas after single therapy using intratumoral CPMV; tumors were collected, cell suspensions obtained and stained using an anti-LAG-3 antibody, and analyzed by flow cytometry on day 1, 5, and 10 post-CPMV intratumoral treatment. Statistical analysis was done by 2-way Anova (****p < 0.0001 CPMV vs. PBS)

**Fig. 4:**
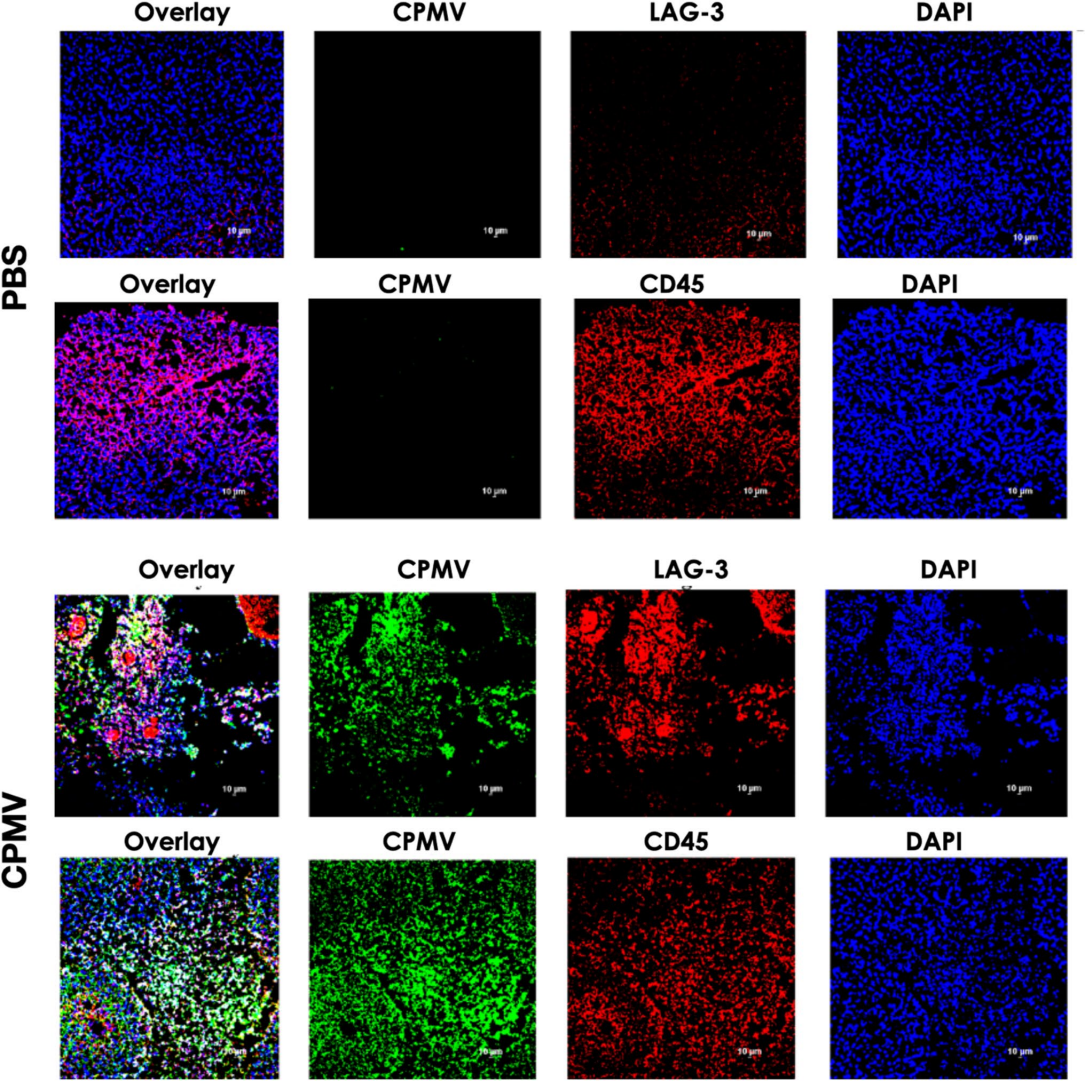
Immunofluorescence staining of B16F10 tumors 10 days post-CPMV or PBS treatment. Tissue sections were stained for CPMV, CD45, and LAG-3

Alteration of the TME with respect to LAG-3 expression was next examined. LAG-3 expression in B16F10 melanomas upon following intratumoral CPMV treatment was examined. Mice-bearing B16F10 dermal melanoma received a single dose of 100 µg intratumoral CPMV. Tumors were collected on day 1, 5, or 10 following CPMV treatment. Single-cell suspensions were obtained and analyzed by flow cytometry. Cryosectioned tissues were analyzed by confocal microscopy. Five and 10 days post CPMV therapy, LAG-3 expression was significantly increased with ~ 44% and 74% of cells, respectively, staining positive for LAG-3 (**** < 0.0001 vs. PBS) (Fig. 3). In addition, tumor sections collected on day 10 post-treatment were stained for immune cells (CD45^+^) and LAG-3. LAG-3 expression was prominently observed in tumors that received CPMV therapy, consistent with the flow cytometry results (Fig. 4).

## Discussion and conclusion

This work represents an initial investigation into a combination immunotherapy strategy employing CPMV IIT and next-generation ICT, with LAG-3 inhibition, to treat B16F10 mouse model of melanoma. We observed increased efficacy with the anti-LAG-3 + CPMV treatment, with more than double the survival rate of CPMV IIT as a single therapy. Anti-LAG-3 treatment alone showed no survival or tumor growth control benefit above the control vehicle group. Flow cytometric analysis of the tumor cell suspension and tumor section immunostaining provided insight into the state of LAG-3 expression among the diverse populations of cells in the TME. This analysis revealed little LAG-3 expression at baseline. With CPMV IIT, however, LAG-3 expression in the TME significantly increased.

ICT has demonstrated remarkable efficacy in treatment of many tumors, albeit often within small subset of patients. As disease recurrence and resistance have arisen with ICT, combination therapies have been increasingly explored to address these limitations. Incorporating multimodal cancer therapies and classic ICT, targeting PD-1/PD-L1 and/or CTL4A, have bolstered efficacy both in preclinical and clinical studies. Multiple clinical investigations of combination approaches incorporating LAG-3 inhibitors have demonstrated encouraging results with favorable safety and tolerability profiles in phase I and II clinical trials [53–56]. Phase III clinical trials in patients with advanced melanoma demonstrated increased progression free survival [57] and high pathologic response rates [58] with combined PD-1/PD-L1 inhibition and LAG-3 inhibition over PD-1/PD-L1 inhibition alone. Similarly, our preclinical investigations have revealed synergy in therapeutic strategies combining CPMV IIT with ICTs [35, 44, 61]. Here, we also extend our investigations to include LAG-3. While beyond the scope of this work—considering the encouraging clinical results using LAG-3 and PD-1 in combination—one could also test CPMV in combination with LAG-3 and PD-1 and compare its efficacy against single combinations using CPMV with either LAG-3 or PD1 blockade.

LAG-3 has broad patterns of expression and contributes to homeostasis and regulation of function of a wide variety of cells, including CD4^+^ T cells, CD8^+^ T cells, NK cells, tumor cells, and pDCs. [45–47] Its expression on pDCs is greater than on any other immune cell subset. pDC plays an important role in induction of immune responses, however, these cells may contribute to immunosuppression in the TME. pDCs expressing LAG-3 have been shown to be activated by interaction with MHCII on human melanoma in vivo*,* leading to IL-6 production, which in this setting could contribute to MDSC recruitment [52]. In addition, it has been suggested that immunogenicity of a tumor determines the degree to which LAG-3 inhibition affects immune responses [47]. Hence, combination with a therapy, like CPMV IIT, that converts a “cold” tumor into a “hot” tumor could be particularly synergistic with LAG-3. Our data indicate that CPMV IIT induces significant expression levels of LAG-3 within the TME—considering that > 70% of the cell suspension from CPMV-treated B16F10 tumor stained positive for LAG-3 vs. approximately 30% for PBS control group (see Fig. 3). These data indicate that multiple cell types may be involved—immune and tumor cells. Prior work has shown that CPMV primarily interacts with immune cells within the tumor, primary responders are neutrophils [25], however, uptake in other phagocyte cells (such as macrophages and DCs) [62] as well as tumor cells themselves has also been documented [63]. The immunofluorescence analysis indicates that B16F10 tumors are infiltrated by immune cells—independent of treatment. However, significant LAG-3 expression is only observed upon CPMV treatment, and the expression pattern of LAG-3 appears to match the localization of CPMV-positive cells. Future functional assays should dissect which cells CPMV interacts with and whether CPMV uptake directly correlates with LAG-3 expression or whether expression is restricted to certain cell types.

Previous studies of CPMV IIT from our research group showed its remodeling of the TME, recruiting innate immune cells and pro-inflammatory cytokine release [26–28]. CPMV IIT also promotes tumor infiltration and activation of antigen presenting cells (APCs) and promotes priming of a systemic, targeted, long-term anti-tumor adaptive immune system response [26, 28]. CPMV’s ability to create a more immunogenic TME may also underlie the synergy of combined intratumoral CPMV and LAG-3 inhibition over LAG-3 inhibition alone. Additionally, combinations of CPMV and PD-1 inhibition, OX40 agonism, and 4-1BB inhibition have further yielded heightened efficacy beyond treatment with either therapy alone [35, 44, 61]. Intratumoral CPMV led to increased expression of PD-1/PD-L1 and OX40 on CD4^+^, CD8^+^ T cells, and Tregs in multiple murine tumor models, including B16F10. [35]

We observed elevated LAG-3 expression in the tumor cell suspension and in immunostained tumor sections. Although an effective anti-tumor immune response was observed with the intratumoral CPMV alone, the efficacy was more than doubled, with respect to survival, by the addition of the anti-LAG-3. This enhanced efficacy is likely related to blockade of the LAG-3, “neutralizing” its increased expression, in the tumor microenvironment following CPMV treatment. Thus, allowing a more potent intratumoral CPMV-primed immune response than that occurring in the absence of anti-LAG-3. A study of patients with metastatic melanoma also observed enhanced expression of LAG-3 following ICT treatment with dual anti-PD-1 and anti-LAG-3 therapy. Interestingly, enhanced LAG-3 expression in dendritic cells and tumor infiltrating lymphocytes (TILs) was significantly greater in patients that responded to therapy over non-responders [64]. Multiple other clinical studies have revealed substantial LAG-3 expression on cells within the TME. [65]

We have previously observed enhanced efficacy of treatment with CPMV and ICTs primarily activating or disinhibiting effector T cells and in the case of 4-1BB, NK cells [35, 44, 61]. Inhibition of the varied expression pattern of LAG-3 could promote a more diverse pro-inflammatory response, involving all these cell types. Combination of these anti-LAG-3 effects with the pro-inflammatory TME transformation and immune response induced by CPMV IIT dramatically increases efficacy against a murine model of melanoma.

## Data Availability

Data will be shared by the authors upon reasonable request.
